# Choriocapillaris Ischemia at the Leakage Point of Patients With Acute Central Serous Chorioretinopathy

**DOI:** 10.3389/fmed.2021.675876

**Published:** 2021-09-07

**Authors:** Zuofen Wang, Zhaoting Xin, Jun Yang, Huawen Lu, Haiying Wang, Lin Zhu

**Affiliations:** ^1^Zibo Central Hospital, Binzhou Medical University, Zibo, China; ^2^Wuzhou Gongren Hospital, Wuzhou, China

**Keywords:** central serous chorioretinopathy, leakage point, choriocapillaris vessel density, optical coherence tomographic angiography, vessel density ratio

## Abstract

**Purpose:** We aimed to determine ischemia of the choriocapillaris at the leakage point of patients with acute central serous chorioretinopathy (CSC) by optical coherence tomographic angiography (OCTA).

**Methods:** A retrospective study of 38 eyes of 38 acute CSC patients with spontaneous complete resolution of subretinal fluid (SRF) was conducted and patients were followed for 3 months. Fundus fluorescein angiography (FFA) and indocyanine green angiography (ICGA) were performed at baseline. Best corrected visual acuity (BCVA) and OCTA were collected at baseline and at follow-up visits. An age- and refractive error-matched control group consisted of 40 eyes of 40 healthy people.

**Results:** The BCVA of patients significantly improved at 1 and 3 months. At baseline, all eyes showed a decreased choriocapillaris vessel density. The mean vessel density of superficial choroid (VDSC) at the leakage point area was 44.18 ± 9.27, which increased to 54.31 ± 9.70 at 1 month (*p* < 0.001) and to 55.19 ± 6.46 at 3 months (*p* < 0.001). The mean vessel density ratio was 0.90 ± 0.16 at baseline, which increased to 0.96 ± 0.15 at 1 month (*p* = 0.037) and to 0.97 ± 0.08 at 3 months (*p* = 0.016). The highest VDSC of patients was lower than that of normal control (*p* < 0.001).

**Conclusions:** The VDSC at the leakage point of acute CSC patients was significantly thinner and regularly increased with the recovery process, which suggested that ischemia might be one of the initiating factors in the pathogenesis of acute CSC.

## Introduction

Central serous chorioretinopathy (CSC) is an idiopathic ophthalmopathy in which the neurosensory retina is often detached in the central macular region because of serous leakage from defects of the retinal pigment epithelium (RPE) (1–4). The increasing use of fundus fluorescein angiography (FFA) and indocyanine green angiography (ICGA) in CSC has greatly improved the understanding of the pathogenesis of CSC and demonstrates that choroidal circulation dysfunction primarily leads to CSC. The pathogenesis of CSC may be related to an alteration of the outer barrier effect and the pumping function of the RPE and an increase in leakage and permeability in the choroidal vasculature (5–7). However, the mechanisms are still unclear because of the complexity of the choroidal microvasculature.

With the development of examination techniques, optical coherence tomography angiography (OCTA) has been developed to generate a volumetric rendering of blood flow in different layers of the retina and choroid, capable of detecting the vascular morphology and vessel density of the retinochoroid capillaries quantitatively without injecting dye (8–12). Some OCTA researches have confirmed the choroidal dysfunctions in patients with CSC (11, 13, 14). The choroid is primarily a vascular structure consisting mostly of blood vessels, which is the source of oxygen and other nutrition for RPE cells. A disordered microvasculature can generate ischemia of the RPE cells. Therefore, we hypothesized that the dysfunction of the extracellular barrier of RPE caused by ischemia may also be an important factor in the initial pathogenesis of CSC.

The purpose of our study was to determine the existence of ischemia at the leakage point of acute CSC with OCTA and investigate whether the ischemia reduced with recovery.

## Methods

This multicenter retrospective clinical study adhered to the tenets of the Declaration of Helsinki and was approved by the institutional review board of Zibo Central Hospital and Wuzhou Gongren Hospital. The Ethics Committee rules that written informed consent was not required because our study was retrospective in nature and all the images were fully anonymized.

Patients with dome-shaped serous detachment of the sensory retina and active leakage on FFA were eligible at baseline. Among whom, those who achieved spontaneous complete resolution of SRF in 3 months were recruited from February 1, 2016 to February 29, 2020. A total of 38 eyes of 38 CSC patients were enrolled and examined at our clinic center. An age- and refractive error-matched control group consisted of 40 eyes of 40 healthy people.

### Inclusion and Exclusion Criteria

Acute CSC was defined as persistent SRF for <6 months (15–17). The following inclusion criteria were fulfilled: (1) patients between 18 and 55 years of age, first episode; (2) the locations of the leakage points are <1,500 μm away from the fovea on FFA; (3) abnormal dilated choroidal vasculature on ICGA; and (4) SRF involving the fovea on OCTA. The exclusion criteria were defined as follows: (1) patients who underwent previous treatment, including photodynamic therapy (PDT), thermal laser photocoagulation, or intravitreal injection of anti-vascular endothelial growth factor (VEGF); (2) patients with other fundus disease such as polypoidal choroidal vasculopathy (PCV), choroidal neovascularization (CNV), and other retinochoroidal vascular diseases and maculopathies; (3) patients with pigment epithelial detachment (PED) at the leakage point with average diameters (transverse diameter and vertical diameter) of more than 300 μm; (4) serous retinal detachment with subretinal accumulation of highly reflective fibrin on OCT; (5) high myopia (refractive diopter more than 6 D); (6) patients receiving treatment of exogenous corticosteroid systemically; (7) pregnancy; and (8) patients with media opacities and inability to perform relative fundus examination, or signal strength index of the images <7.

### Ophthalmic Examinations

Each patient underwent a baseline assessment and subsequent examinations at the 1- and 3-month follow-up visits. Baseline assessment comprised best corrected visual acuity (BCVA) measurement, intraocular pressure by non-contact tonometer, and dilated fundus biomicroscopy. Furthermore FFA (Heidelberg, Germany), ICGA (Heidelberg, Germany), and OCTA using the split-spectrum amplitude-decorrelation angiography algorithm (Optovue, Fremont, CA, USA) were also performed. Each follow-up examination included BCVA and OCTA. In order to avoid as much as possible the influence of the body's daily hormone levels on retinochoroidal circulation and to minimize the influence of time on the vessel conditions of the retinochoroid, the examinations were conducted in the morning between 8:00 a.m. and 12:00 a.m. at each follow-up visit.

### Optical Coherence Tomography Angiography

**Figure 1:**
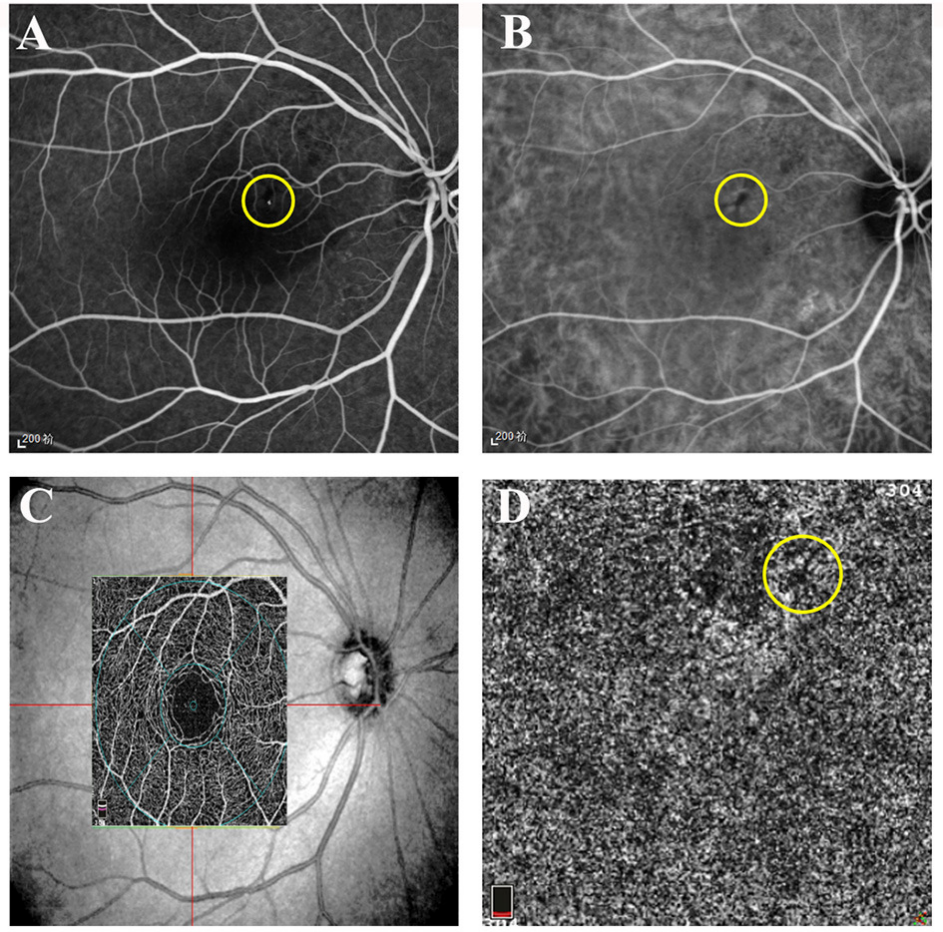
Representative images of active central serous chorioretinopathy (CSC). **(A)** Early-phase fundus fluorescein angiography (FFA) of the right eye of a 39-year-old female patient with acute CSC. A hyperfluorescent superofoveal was visible and marked by a *yellow circle*. **(B)** Early-phase indocyanine green angiography (ICGA) of the same patient showing a hypofluorescent area and marked by a *yellow circle*. **(C)** The superficial retina layer and the corresponding location of the 3 × 3 image on optical coherence tomographic angiography (OCTA). **(D)** The low signal area at the leakage point corresponding to FFA and ICGA.

OCTA was performed at baseline and at each follow-up visit. The images were displayed at four different layers using Optovue software (version 2018.0.0.13): superficial, deep, outer retina layers, and superficial choroid layer. Images of the superficial choroid layer were used to observe changes in the area of the leakage point and the subsequent changes in the follow-up visits. The superficial retina was defined as the inner limiting membrane (ILM) to 10 μm above the inner plexiform layer (IPL). The deep retina was defined as 10 μm above the IPL to 10 μm below the outer plexiform layer (OPL). In general, the superficial and deep retina layers were considered as the inner retina. The outer retina was defined as 10 μm below the OPL to 10 μm above the Bruch's membrane (BRM); less emphasis was placed on the outer retina as, theoretically, there was an absence of capillaries. The superficial choroidal capillary layer was defined as 10 μm above the BRM to 30 μm below the BRM. In this study, we focused on the analysis of the vessel density of the superficial choroid (VDSC) in patients with CSC. The 3-mm × 3-mm scanning pattern centered on the fovea was used. We analyzed the average VDSC and the VDSC just below the leakage point shown on FFA (Figure 1).

### Follow-Up Protocol and Outcome Measures

Patients were seen for regular follow-up at 1 and 3 months after the first visit. A standardized evaluation was performed at each visit, including BCVA at 4 m, according to the guidelines of the Early Treatment Diabetic Retinopathy Study (ETDRS), ophthalmoscopy, and OCTA. The primary outcome measures were the changes of the choriocapillaris in affected eyes in OCTA, which was measured as the vessel density at the leakage point and the vessel density ratio of the leakage point to the whole area. Secondary outcomes were the complete absorption rate of SRF based on OCTA images at 3 months and changes of the BCVA.

### Vessel Density Ratio

**Figure 2:**
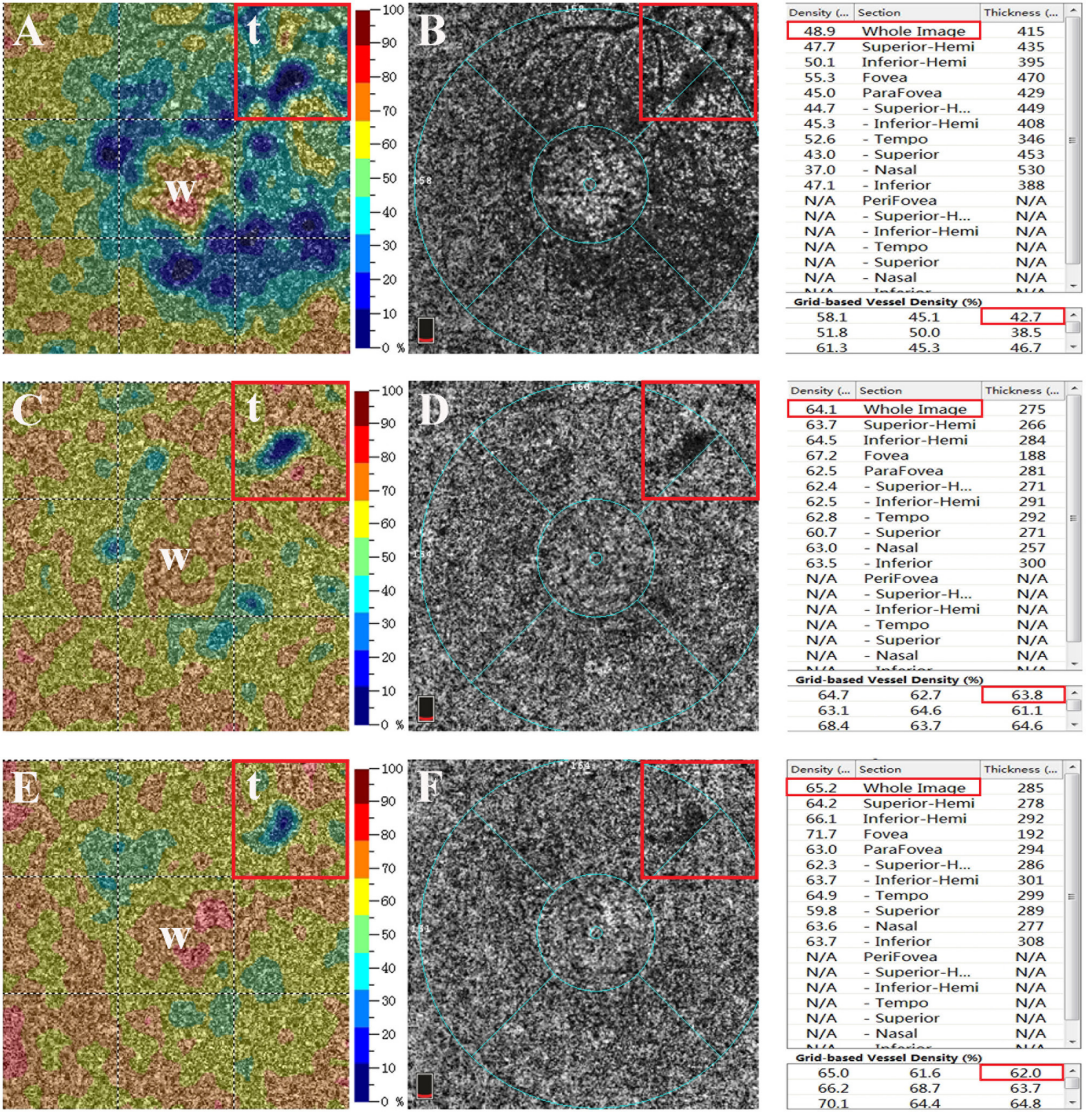
Superficial choroidal layer on optical coherence tomographic angiography (OCTA) image. **(A,B)** The mean vessel densities of the whole area (area “*w*” in figure) and the target area (area “*t*” in figure) were measured (Vt and Vw, respectively). The mean vessel density ratio was calculated at each follow-up visit (vessel density ratio = Vt/Vw). **(C,D)** OCTA image of the superficial choroidal layer at the 1-month follow-up visit. **(E,F)** OCTA image of the superficial choroidal layer at the 3-month follow-up visit. The *right panel* shows the mean vessel density of the superficial choroidal layer at baseline and at 1 and 3 months and the mean vessel density in each subregion. The *red box* marks the vessel density of the whole area and the target area at the leakage point.

The image of the superficial choroidal layer shown by OCTA was used to analyze the changes of choriocapillaris in affected eyes, and the vessel density was calculated as the percentage of pixels with a flow signal greater than the threshold (Figure 2) (18–20). We analyzed the vessel density of the leakage point and then calculated the vessel density ratio of the superficial choroidal layer on OCTA pictures. An example of the calculation of the vessel density ratio is depicted in Figure 2. Firstly, an image of the superficial choroidal layer was selected in the eye with acute CSC that was planned to be enrolled. Secondly, the vessel density of the target area was marked out by the relative position of the vessels on FFA and the distance to the fovea measured by OCTA. The mean density of the choriocapillaris in the target area (area “*t*” in Figure 2) and the whole image (area “*w*” in Figure 2) were measured (Vt and Vw, respectively). Thirdly, the mean vessel density ratio of the target to the whole area was calculated at each follow-up (“vessel density ratio = Vt/Vw”) (Figure 2). Then, we compared the vessel density ratios at baseline and at the 1- and 3-month follow-up visits.

### Statistical Analyses

All data were presented as the mean ± standard deviation (SD). The Shapiro–Wilk test was used to examine the normal distribution of data. Serial comparisons of the mean BCVA, VDSC, and vessel density ratio were conducted with a paired samples *t*-test. Additionally, an independent samples *t*-test was performed to compare the VDSC among CSC eyes, fellow eyes, and normal eyes. Statistical analyses were carried out with SPSS software, version 19 (IBM, Armonk, New York, USA). A *p*-value < 0.05 was considered statistically significant.

## Results

### Baseline Demographic Data and Improvement of BCVA

A total of 38 eyes of 38 CSC patients were enrolled in the study. The average duration before enrollment was 3.53 ± 1.48 months. The follow-up visits last for longer than 3 months. No patient developed severe side effects related to the surgery, such as severe vision loss, RPE atrophy, or development of CNV. Demographic data are shown in Table 1. The mean BCVA was 66.05 ± 8.20 letters on the ETDRS chart at baseline assessment and significantly improved to 75.79 ± 4.92 and 80.13 ± 3.55 letters at 1 and 3 months, respectively (Table 2).

**Table 1:** Baseline demographic data of the patients.

**Characteristics**	**Patients**	**Control group**
Patients (male/female)	35/3	36/4
Eyes, *n*	38	40
Age (years)	45.29 ± 6.52	44.66 ± 4.87
Duration of symptom (months)	3.53 ± 1.48	0
BCVA (letters, ETDRS)	66.05 ± 8.20	≥80

*Values are the mean ± SD*.

*BCVA, best corrected visual acuity; ETDRS, early treatment diabetic retinopathy study*.

**Table 2:** BCVA and vessel density of the superficial choroidal capillaries of CSC patients.

**Characteristics**	**Baseline**	**1 month**	***p*-value (baseline vs. 1 month)**	**3 months**	***p*-value (baseline vs. 3 months)**
BCVA (letters)	66.05 ± 8.20	75.79 ± 4.92	<0.001^*^	80.13 ± 3.55	<0.001^*^
VDSCw (%)	49.05 ± 6.63	56.57 ± 5.83	<0.001^*^	56.87 ± 5.75	<0.001^*^
VDSCt (%)	44.18 ± 9.27	54.31 ± 9.70	<0.001^*^	55.19 ± 6.46	<0.001^*^
Vt/Vw	0.90 ± 0.16	0.96 ± 0.15	0.037^*^	0.97 ± 0.08	0.016^*^

*Values are shown as the mean ± SD*.

*BCVA, best corrected visual acuity; CSC, central serous chorioretinopathy; VDSCw, vessel density of the superficial choroid (calculated as the percentage of pixels with a flow signal greater than the threshold); VDSRt, vessel density of the superficial choroid at the target area; Vt/Vw, ratio of the mean vessel density of the superficial choroid in the target area (area “t” in Figure 2) and the whole image (area “w” in Figure 2)*.

* *p < 0.05 (statistically significant difference)*.

### Changes of Superficial Choroidal Capillaries

**Figure 3:**
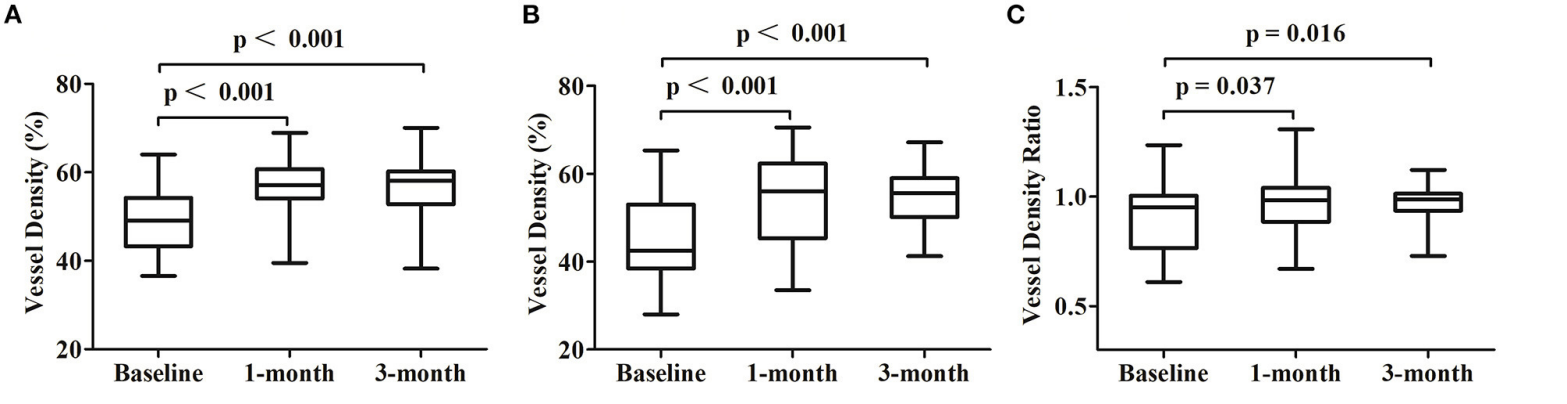
Changes of the optical coherence tomography angiography parameters at each follow-up visit. **(A)** Comparisons of the vessel density at the whole area of the superficial choroidal layers. **(B)** Comparisons of the vessel density at the target 3 × 3 area of the superficial choroidal layers. **(C)** Comparisons of the vessel density ratio of the superficial choroidal layers.

The mean VDSC was 49.05 ± 6.63 before 577-nm subthreshold micropulse macular laser (SML), which increased to 56.57 ± 5.83 at 1 month and 56.87 ± 5.75 at 3 months (*p* < 0.001 and *p* < 0.001, respectively). The mean vessel density of the target area where the leakage point is located was 44.18 ± 9.27 at baseline and then increased to 54.31 ± 9.70 at 1 month and to 55.19 ± 6.46 at 3 months (*p* < 0.001 and *p* < 0.001, respectively) (Table 2; Figures 2, 3).

The mean vessel density ratio was 0.90 ± 0.16 at baseline, which increased to 0.96 ± 0.15 at 1 month and to 0.97 ± 0.08 at 3 months (*p* = 0.037 and *p* = 0.016 respectively) (Table 2; Figures 2, 3).

### Comparison With Fellow Eyes and Normal Eyes

**Figure 4:**
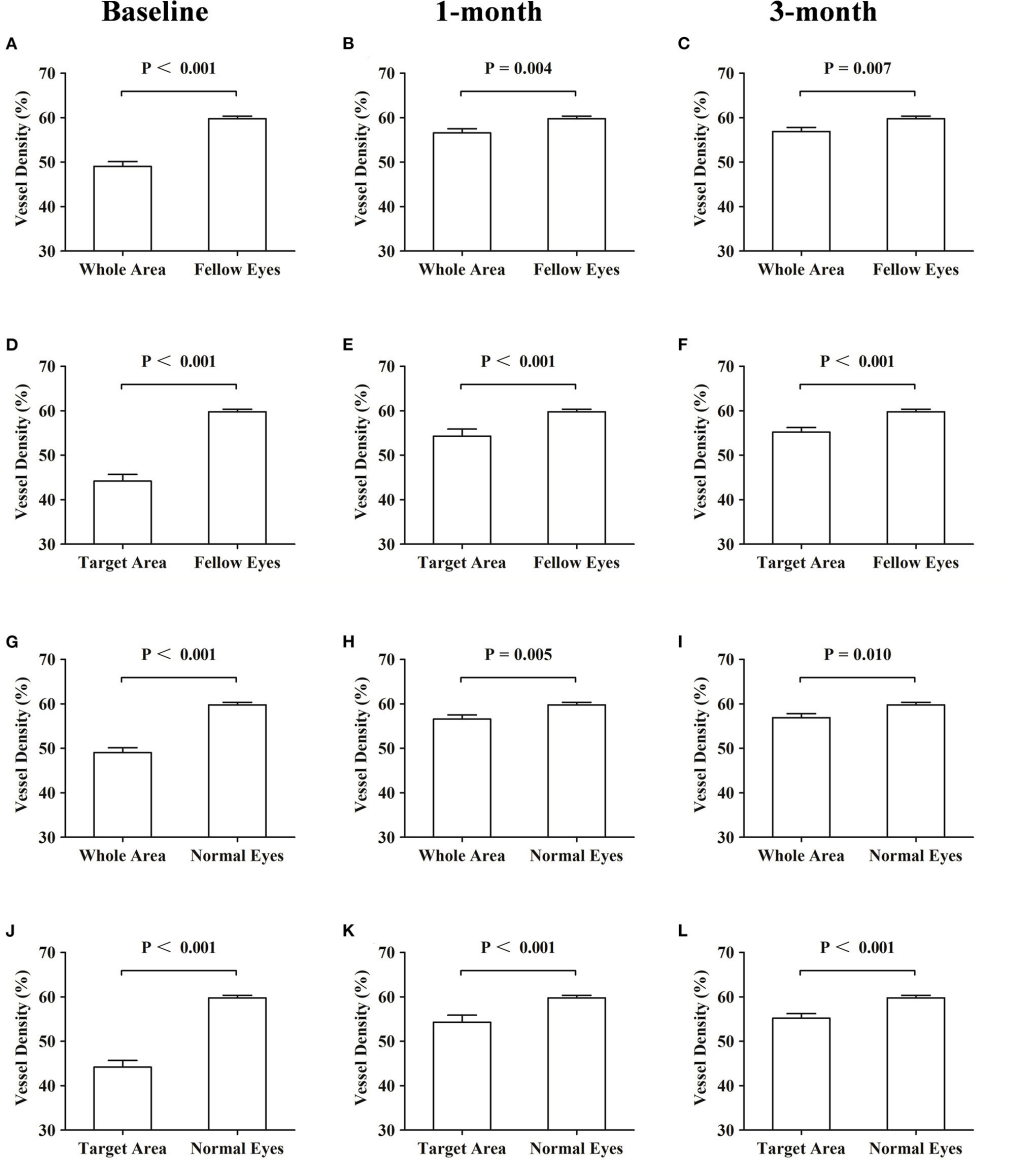
Comparisons of the vessel density of the superficial choroidal layers at baseline and at 1 and 3 months. Comparisons of the vessel density of the superficial choroidal layers. **(A–C)** Whole area vs. fellow eyes. **(D–F)** Target area vs. fellow eyes. **(G–I)** Whole area *vs*. normal eyes. **(J–L)** Target area vs. normal eyes.

CSC patients were similar to the normal control in age and sex distribution. The mean VDSC of affected eyes at baseline assessment was significantly lower compared to those of fellow eyes and normal eyes (*p* < 0.001 and *p* < 0.001, respectively). The VDSC increased significantly with complete absorptions of SRF at 3 months. However, the VDSC value was still lower than those of fellow eyes and normal eyes, and the difference was statistically significant (*p* = 0.041 and *p* = 0.031, respectively). There was little difference in the VDSC between normal eyes and fellow eyes (Table 3; Figure 4).

**Table 3:** Comparisons of affected eyes, fellow eyes, and normal control at baseline.

**Characteristics**	**VDSC (%)**	***p*-value (A vs. N)**	***p*-value (A vs. F)**
Whole area (baseline)	49.05 ± 6.63	<0.001^*^	<0.001^*^
Target area (baseline)	44.18 ± 9.27	<0.001^*^	<0.001^*^
Whole area (1 month)	56.57 ± 5.83	0.004^*^	0.005^*^
Target area (1 month)	54.31 ± 9.70	<0.001^*^	<0.001^*^
Whole area (3 months)	56.87 ± 5.75	0.007^*^	0.010^*^
Target area (3 months)	55.19 ± 6.46	<0.001^*^	<0.001^*^
Fellow eyes	59.77 ± 3.54	N/A	N/A
Normal eyes	60.05 ± 4.19	N/A	N/A

*Values are shown as the mean ± SD*.

*VDSC, vessel density of superficial choroid; A, affected eyes; F, fellow eyes; N, normal eyes*.

* *p < 0.05 (statistically significant difference)*.

## Discussion

Acute CSC is a posterior segment disease that represents a common cause of vision loss in the middle-aged population (4, 7). Currently, FFA and ICGA remain the golden standard for the diagnosis of CSC, but their application is limited due to being invasive and requiring intravenous administration of dye (5). The updated OCTA is an easy and non-invasive method to visualize intravascular flow at the microcirculation level in 5–6 s, and observation of the superficial choroidal capillaries in a three-dimensional perspective is more intuitive (21). We quantitatively evaluated and analyzed the characteristics of the superficial choriocapillaris at the leakage point area of acute CSC eyes using OCTA images for the first time.

The results of our study demonstrated that the VDSC was significantly lower in eyes with acute CSC than in fellow eyes and in age- and refractive error-matched normal eyes, and the decrease of the vessel density was more obvious at the leakage point on OCTA images. In order to eliminate the influence of interfering factors (mainly referring to SRF) as much as possible, we calculated the vessel density ratio at the leakage point area of the superficial choroidal layer on OCTA pictures. The mean VDSC at the leakage point area and the whole 3 × 3 OCTA images both increased at 1 and 3 months, and the ratio of VDSC also increased at the follow-up visits. However, the VDSC was still lower than that of the normal control, and the difference was statistically significant. Our research also demonstrated that the pathogenesis of acute CSC was a consequence of a choroidal disease, and the decreased choroidal vessel density had a regeneration with the recovery of CSC.

At baseline, the low vessel density ratio indicates a more severe ischemia at the leakage points. Scheider et al. (22) had confirmed the reduction in choroidal blood flow in CSC eyes and demonstrated that the hypoperfusion persisted after clinical improvement with combined FFA/ICGA imaging in 1993. Although the etiology and the pathogenesis of CSC are still to be determined, previous studies using ICGA have shown that the choroid is primarily involved in the pathogenesis of CSC, the abnormalities of CSC eyes including delayed filling, vascular congestion, choroidal vascular hyperpermeability, and punctate hyperfluorescent spots (23–27). On OCTA, choriocapillary hypoperfusion and hyperperfusion often coexist in the abnormal vascular areas of ICGA and the leakage point area of FFA (11, 14, 18). Some researchers have proposed the hypothesis that the hypoperfusion with hyperperfusion in the surrounding area on OCTA may indicate the presence of ischemic factors in the corresponding area (11, 13, 21), and our research proves this hypothesis in patients with acute CSC quantitatively.

To the best of our knowledge, this is the first retrospective study to quantitatively investigate the vessel density ratio of the superficial choroidal layer at the leakage point of patients with acute CSC using OCTA. A previous study by Chan et al. (14, 28) has shown that the high signal intensity of the choriocapillaris on OCTA is more common in chronic CSC. However, according to our data, a reduced blood signal intensity in the superficial choriocapillaris layer of OCTA occurs more frequently in acute CSC eyes, which corresponds to the location of the leakage point on FFA, and most of them are not surrounded by high signals, as described in chronic CSC eyes. This phenomenon indicates that a high signal intensity in chronic CSC may be a compensatory mechanism of the choroid due to early ischemia. Given that the microcirculation of the choroidal capillary probably becomes more complicated, early intervention is necessary to reduce the possibility of developing severe anatomic and functional damage. The vessel density ratio of VDSC in OCTA decreased significantly at baseline and increased during subsequent follow-up visits, which also indicated that ischemic factors play an important role in the pathogenesis of acute CSC. The dysfunction and atrophy of the photoreceptor and RPE were connected to a decreased nutrition supply, which may lead to impairment of the RPE cell tight junction and RPE barrier breakdown and RPE/sensory retinal detachment. With the recovery of disease, the vessel density ratio of VDSC improved significantly and the ischemic status also ameliorated obviously, which may be an important factor in the recovery of the tight connection function of RPE cells.

The limitations of this study are related to the small number of patients and short follow-up time. A further expanded sample size and prolonged follow-up can help to confirm our conclusion. Although patients with PED were excluded from this study by average diameters >300 μm, the presence of undetected PED would create an imperceptible “dark area” on the superficial choroidal layer, leading to underestimations of the vessel density. In addition, the effect of SRF was excluded by calculating the vessel density ratio in our research, and the result might be biased due to the different heights of SRF.

In conclusion, our research revealed that the vessel density of the choroidal capillary layer was significantly thinner at the leakage point of acute CSC, and the choroidal capillary tended to regenerate with recovery. This phenomenon suggests that ischemia may be an important factor in the pathogenesis and the recovery of acute CSC.

## Data Availability Statement

The raw data supporting the conclusions of this article will be made available by the authors, without undue reservation.

## Ethics Statement

This multiple-center retrospective clinical study was adhered to the tenets of the Declaration of Helsinki and approved by the institutional review board of Zibo Central Hospital and Wuzhou Gongren Hospital. The patients/participants provided their written informed consent to participate in this study.

## Author Contributions

ZW and LZ conceptualized and designed the study. LZ provided administrative support. ZW, ZX, and JY provided the study materials or patients. ZW, ZX, and HL collected and assembled the data. ZX, HL, and JY contributed to data analysis and interpretation. All authors contributed to the article and approved the submitted version.

## Conflict of Interest

The authors declare that the research was conducted in the absence of any commercial or financial relationships that could be construed as a potential conflict of interest.

## Publisher's Note

All claims expressed in this article are solely those of the authors and do not necessarily represent those of their affiliated organizations, or those of the publisher, the editors and the reviewers. Any product that may be evaluated in this article, or claim that may be made by its manufacturer, is not guaranteed or endorsed by the publisher.

## References

[1] Wang M Munch IC Hasler PW Prunte C Larsen M. Central serous chorioretinopathy. Acta Ophthalmol. (2008) 86:126–45. 10.1111/j.1600-0420.2007.00889.x 17662099

[2] Piccolino FC de la Longrais RR Ravera G Eandi CM Ventre L Abdollahi A. The foveal photoreceptor layer and visual acuity loss in central serous chorioretinopathy. Am J Ophthalmol. (2005) 139:87–99. 10.1016/j.ajo.2004.08.037 15652832

[3] Yannuzzi LA. Central serous chorioretinopathy: a personal perspective. Am J Ophthalmol. (2010) 149:361–3. 10.1016/j.ajo.2009.11.017 20172062

[4] Gemenetzi M De Salvo G Lotery AJ. Central serous chorioretinopathy: an update on pathogenesis and treatment. Eye. (2010) 24:1743–56. 10.1038/eye.2010.130 20930852

[5] Ting DSW Cheung CY Lim G Tan GSW Quang ND Gan A. Development and validation of a deep learning system for diabetic retinopathy and related eye diseases using retinal images from multiethnic populations with diabetes. JAMA. (2017) 318:2211–23. 10.1001/jama.2017.18152 29234807 PMC5820739

[6] van Rijssen TJ van Dijk EHC Dijkman G Boon CJF. Clinical characteristics of chronic central serous chorioretinopathy patients with insufficient response to reduced-settings photodynamic therapy. Graefes Arch Clin Exp Ophthalmol. (2018) 256:1395–402. 10.1007/s00417-018-4003-z 29732468 PMC6060777

[7] Min JY Lv Y Yu S Gong YY. Findings of OCT-angiography compared to fluorescein and indocyanine green angiography in central serous chorioretinopathy. Lasers Surg Med. (2018) 50:987–93. 10.1002/lsm.22952 29896889

[8] Golebiewska J Brydak-Godowska J Moneta-Wielgos J Turczynska M Kecik D Hautz W. Correlation between choroidal neovascularization shown by OCT angiography and choroidal thickness in patients with chronic central serous chorioretinopathy. J Ophthalmol. (2017) 2017:3048013. 10.1155/2017/3048013 29109866 PMC5646334

[9] Peiretti E Iovino C Sacconi R Caminiti G Querques G. Optical coherence tomography angiography characteristics of polypoidal choroidal vasculopathy secondary to chronic central serous chorioretinopathy. Retina. (2019) 39:1693–1700. 10.1097/IAE0000000000002234 29965937

[10] Sahoo NK Mishra SB Iovino C Singh SR Munk MR Berger L. Optical coherence tomography angiography findings in cystoid macular degeneration associated with central serous chorioretinopathy. Br J Ophthalmol. (2019) 103:1615–618. 10.1136/bjophthalmol-2018-313048 30602447

[11] Teussink MM Breukink MB van Grinsven MJ Hoyng CB Klevering BJ Boon CJ. OCT angiography compared to fluorescein and indocyanine green angiography in chronic central serous chorioretinopathy. Invest Ophthalmol Vis Sci. (2015) 56:5229–37. 10.1167/iovs.15-17140 26244299

[12] Bonini Filho MA de Carlo TE Ferrara D Adhi M Baumal CR Witkin AJ. Association of choroidal neovascularization and central serous chorioretinopathy with optical coherence tomography angiography. JAMA Ophthalmol. (2015) 133:899–906. 10.1001/jamaophthalmol.2015.1320 25996386 PMC4721607

[13] Cakir B Reich M Lang S Buhler A Ehlken C Grundel B. OCT angiography of the choriocapillaris in central serous chorioretinopathy: a quantitative subgroup analysis. Ophthalmol Ther. (2019) 8:75–6. 10.1007/s40123-018-0159-1 30617944 PMC6393260

[14] Chan SY Wang Q Wei WB Jonas JB. Optical coherence tomographic angiography in central serous chorioretinopathy. Retina. (2016) 36:2051–8. 10.1097/IAE.0000000000001064 27164548

[15] Breukink MB Mohr JK Ossewaarde-van Norel A den Hollander AI Keunen JE Hoyng CB. Half-dose photodynamic therapy followed by diode micropulse laser therapy as treatment for chronic central serous chorioretinopathy: evaluation of a prospective treatment protocol. Acta Ophthalmol. (2016) 94:187–97. 10.1111/aos.12938 26670630

[16] Lai TY Chan WM Li H Lai RY Liu DT Lam DS. Safety enhanced photodynamic therapy with half dose verteporfin for chronic central serous chorioretinopathy: a short term pilot study. Br J Ophthalmol. (2006) 90:869–74. 10.1136/bjo.2006.090282 16597666 PMC1857171

[17] van Rijssen TJ van Dijk EHC Yzer S Ohno-Matsui K Keunen JEE Schlingemann RO. Central serous chorioretinopathy: towards an evidence-based treatment guideline. Prog Retin Eye Res. (2019) 73:100770. 10.1016/j.preteyeres.2019.07.003 31319157

[18] Feucht N Maier M Lohmann CP Reznicek L. OCT angiography findings in acute central serous chorioretinopathy. Ophthalmic Surg Lasers Imaging Retina. (2016) 47:322–7. 10.3928/23258160-20160324-03 27065370

[19] Yu J Jiang C Wang X Zhu L Gu R Xu H. Macular perfusion in healthy chinese: an optical coherence tomography angiogram study. Invest Ophthalmol Vis Sci. (2015) 56:3212–7. 10.1167/iovs.14-16270 26024105 PMC4455309

[20] Agemy SA Scripsema NK Shah CM Chui T Garcia PM Lee JG. Retinal vascular perfusion density mapping using optical coherence tomography angiography in normals and diabetic retinopathy patients. Retina. (2015) 35:2353–63. 10.1097/IAE.0000000000000862 26465617

[21] Rochepeau C Kodjikian L Garcia MA Coulon C Burillon C Denis P. Optical coherence tomography angiography quantitative assessment of choriocapillaris blood flow in central serous chorioretinopathy. Am J Ophthalmol. (2018) 194:26–34. 10.1016/j.ajo.2018.07.004 30053475

[22] Scheider A Nasemann JE Lund OE. Fluorescein and indocyanine green angiographies of central serous choroidopathy by scanning laser ophthalmoscopy. Am J Ophthalmol. (1993) 115:50–6. 10.1016/S0002-9394(14)73524-X 8420378

[23] Hayashi K Hasegawa Y Tokoro T. Indocyanine green angiography of central serous chorioretinopathy. Int Ophthalmol. (1986) 9:37–41. 10.1007/BF00225936 3721709

[24] Prunte C Flammer J. Choroidal capillary and venous congestion in central serous chorioretinopathy. Am J Ophthalmol. (1996) 121:26–34. 10.1016/S0002-9394(14)70531-8 8554078

[25] Giovannini A Scassellati-Sforzolini B D'Altobrando E Mariotti C Rutili T Tittarelli R. Choroidal findings in the course of idiopathic serous pigment epithelium detachment detected by indocyanine green videoangiography. Retina. (1997) 17:286–93. 10.1097/00006982-199717040-00002 9279943

[26] Iida T Kishi S Hagimura N Shimizu K. Persistent and bilateral choroidal vascular abnormalities in central serous chorioretinopathy. Retina. (1999) 19:508–12. 10.1097/00006982-199919060-00005 10606450

[27] Tsujikawa A Ojima Y Yamashiro K Ooto S Tamura H Nakagawa S. Punctate hyperfluorescent spots associated with central serous chorioretinopathy as seen on indocyanine green angiography. Retina. (2010) 30:801–9. 10.1097/IAE.0b013e3181c72068 20094008

[28] Gawecki M Jaszczuk-Maciejewska A Jurska-Jasko A Kneba M Grzybowski A. Impairment of visual acuity and retinal morphology following resolved chronic central serous chorioretinopathy. BMC Ophthalmol. (2019) 19:160. 10.1186/s12886-019-1171-5 31345183 PMC6659242

